# A prospective clinical study to understand the factors affecting healing of intestinal primary repair and anastomosis at a tertiary care centre

**DOI:** 10.6026/973206300223728

**Published:** 2026-06-30

**Authors:** Akhilesh Ratnakar, Shikha Pandey, Luv Gupta, Aditi Kumari, Talha Saad, Sheela Jain, Deepak Shrivastava

**Affiliations:** 1 Department of General Surgery, Bundelkhand Government Medical College, Sagar, Madhya Pradesh, India; 2 Department of Obstetrics & Gynaecology, Bundelkhand Medical College Sagar, Madhya Pradesh, India; 3 Department of Respiratory Medicine, Bundelkhand Medical College Sagar, Madhya Pradesh, India

**Keywords:** Anastomosis, intestinal repair, anastomotic leak (AL)

## Abstract

One of the greatest worrisome negative consequences via intestinal anastomosis is anastomotic leak (AL). Therefore, it is of interest to evaluate the risk components of intestinal resection and anastomosis. AL appears higher in older age group that is above 50 years and with higher BMI (generally obese type I). Conditions like diabetes, hypertension, benign and malignant lesions of intestine and type of procedure (routine and emergency) resulted in increased incidence of AL. Thus, data shows the clinical and surgical characteristics linked with anastomotic leak, which may improve pre-operative risk assessment, perioperative management and post-operative healing after intestinal repair and anastomosis.

## Background:

Anastomosis is a Greek word; connotation is 'without a mouth'. The familiar operative procedure which is carried out as emergency and routine procedure is intestinal anastomosis [1]. Individuals having inflammatory bowel disease condition are the most likely to develop anastomotic leaks (AL). AL is also linked for causing abscess in pelvic region which may be result in infection and hematoma formation. AL is the biggest worrisome reason for long inpatient duration resulting in higher cost, heightened chances of morbidity and death rates [2, 3]. Inaccurate procedural technique, hypoperfusion of intestine at suture line, heightened tension at anastomosis and mesentery, infection at treated site and presence of obstruction far away from anastomosis are the multitude elements resulting in causation of AL [4]. The progression of AL is related to older individuals, low haemoglobin values, poor nutritional status with various pre-existing disorders, taking steroids in greater amounts and after having sessions with chemotherapeutic agents. Other elements carrying risk of developing AL are smoking status, increased weight, alcohol abuse, lengthy surgical procedure, blood transfusion during the operative procedure [5]. Intestinal anastomosis reinstates interaction among two formerly detached intestinal portions by reinstating their seamless integration. A troublesome portion of the colon is additionally eliminated during the surgical process. It is a typical medical surgery that is carried undertaken both as a matter of urgency and as a personal choice [6]. AL prevalence varies between 0.5% to 30% in the published literature, whereas it frequently appears to be among 2% and 5%. It generally appears between the third- and sixth-day subsequent operations. AL mortality is still high, with estimates ranging from 10% to 30%. The warning signs that trigger AL are precisely the same as those that forecast wound dehiscence considering anastomotic recovery relies on the same principles as the healing of wounds. AL trigger may be segregated into two categories: host-linked elements and surgical procedure-associated variables. In contrast, once the fundamental guidelines of gastrointestinal closure are adhered to, there is barely any distinction across the consequences of anastomoses executed by trainees and those executed by experienced surgeons pursuant to adequate guidance [7, 8]. Therefore, it is of interest to assess the composite variables impacting the anastomosis intervention linked with AL in hospital setting.

## Materials and Methods:

## Research design and sample:

The present research was a prospective clinical investigation which was performed on 82 patients. Subjects older than 20 years, necessitating resection of the intestine and anastomosis (either emergency or routine operations) were the inclusions. Subjects who were recommended repair of intestinal perforation, trauma to abdomen and stoma closure were included in the research. Subjects age less than 20 years old, anastomosis at various sites, biliary enteric anastomosis, anastomosis via stapler, previous history of AL and unable to give informed consent were the exclusions of the present research. The prevalence rate of AL is about 30% as per the literature [1]. Assuming 10% margin of error at 95% confidence interval, value of N is 81. A detailed history taking, clinical examination and haematological and radiological evaluation was done before undergoing the procedure.

## Ethical considerations:

Ethical clearance was obtained before starting the study. Every safety measure was implemented to ensure that research participants' personal information remained private and protected. The ideals of the Declaration of Helsinki guided our research. Every subject gave their informed consent.

## Data collection:

Various parameters were analysed. Demographic data was recorded which comprised of patient age (categorised into <20 years, 21-35 years, 36-50 years and >51 years), gender (male and female), BMI (Underweight, Normal, Overweight, Obese type I and Obese type II) and smoking status (Never smoked, former smoker and current smoker). Data collection also included co-morbid conditions, suture tension (tight, loose, adequate), biochemical abnormalities, type of procedure (Routine or emergency), Operative procedure techniques and complications after surgical procedure (AL, septicaemia, acute renal failure ARF, infection at site of surgery, complications related to respiratory system and Burst abdomen).

## Statistical analysis:

Descriptive analysis was computed by employing mean, standard deviation, frequency and percentage. A chi-square test was implemented to compute the other variables. Analysis was performed using SPSS software.

## Results:

Anastomotic leaks were substantially more common in patients with comorbidities, especially diabetes mellitus (χ^2^=25.1, p<0.001), than in patients without these conditions. Similarly, there was a significant correlation between the frequency of leaks and malignant intestinal lesions compared to benign lesions (80.0% vs. 26.0%, χ^2^=6.6, p=0.01). Additionally, compared to normal surgeries, emergency procedures had a considerably greater leak rate (40.7% vs. 7.1%, χ^2^=10.0, p=0.001), suggesting that post-operative healing results are influenced by both patient-related and disease-related aspects. Conversely, there was no significant correlation found between anastomotic leaks and biochemical indicators such as low hemoglobin, hypoalbuminemia, and altered creatinine levels (p=0.16). Similarly, leak occurrence was not substantially affected by the type of surgical anastomotic approach (side-to-side, end-to-end, or end-to-side) (χ^2^=1.9, p=0.38). These results imply that preoperative biochemical status or the particular surgical technique used are not as significant predictors of anastomotic leakage as concomitant diseases, malignant pathology, and emergency surgical intervention as seen in Table 1.

## Discussion:

Due to the different elements that dictate the course of healing, intestinal anastomosis recuperation is arduous. A method of operation called intestinal anastomosis utilizes to determine contact among two formerly segregated sections of the colon. After an obstruction affecting the bowel is eradicated, this operation refurbishes intestinal continuity. Notwithstanding the actuality that small bowel anastomosis is often executed, notably in a crisis, there is a dearth of research on the recovery process and danger signs that trigger AL. In the present research, patient aged above 50 years exhibited higher incidence of AL and these findings are in line with the observations by Schiff *et al.* [9]. Males exhibited more AL than female subjects in the current research which is similar to the findings observed by several studies [7, 10, 11]. In the present research, we discovered that 63.6% of diabetic subjects had AL and 19.2% of hypertensive subjects had AL and these observations are in line with the results obtained by Telem *et al.* and Shanker *et al.* respectively in their research [12, 13]. Subjects suffering from renal and cardiac disease exhibited higher AL, these results are equivalent to the results reported by Wang *et al.* [8] Patients who were current smokers and greater value of BMI (specifically obese type I) had higher AL in the present study which is alike to the research reported by Wang *et al.* [8]. In the present research, AL was greater in lower haemoglobin, serum albumin and creatinine subjects but it was not noteworthy statistically. Lower haemoglobin causes diminished blood supply to the tissues resulting in ischaemia which can be a reason of AL. These findings are consistent with the similar studies [6, 8, 13]. In our study, 80% patients had AL who was suffering from malignant lesion of intestine. Emergency surgeries encountered greater incidences of AL than routine procedures in our research which is consistent with the outcomes of the research performed by other studies [14, 15]. In the present study, loose type of sutures had greater AL which is in harmonious to the findings of Wang *et al.* [8] in the current research, end to end type procedure had more AL which is alike with findings discovered by Telem *et al.* [12]. In the present study, we discovered that surgical complications like infection, septicaemia, ARF, respiratory problems and burst abdomen suffered by the subjects is homogenous to the findings reported by Bardají-Carrillo *et al.* and Rohit *et al.* [16, 17].

## Conclusion: 

We show that risk components influence healing after intestine primary repair and anastomosis. Anastomotic leaking was linked to both patient-related and surgical factors, emphasizing the necessity of identifying and mitigating risk factors before surgery whenever possible. In high-risk or emergency situations, preventative procedures including diversion ileostomy, colostomy, or drain insertion may assist reduce postoperative problems.

## Figures and Tables

**Table 1:** Statistical correlation among various variables

**Variables**		**No Leaks**	**Leaks**	**Chi square statistic**	**P value**
Comorbidities	Diabetes mellitus (N=22)	8	14	25.1	0.000*
	Hypertension (N=26)	21	5		
	Cardiac disease (N = 3)	1	2		
	Renal disease (N = 4)	2	2		
	No disease (N = 27)	26	1		
Biochemical profile	Haemoglobin <12g/dL (N = 53)	35	18	5.07	0.16 (NS)
	Albumin <3g/dL (N = 21)	18	3		
	Creatinine <0.5mg/dL (N = 3)	1	2		
	Normal biochemical profile (N = 5)	4	1		
Benign and Malignant lesion of intestine	Benign (N = 77)	57	20	6.6	0.01*
	Malignant (N = 5)	1	4		
Procedure	Routine (N = 28)	26	2	10	0.001*
	Emergency (N = 54)	32	22		
Surgical Techniques	End to End (N = 61)	41	20	1.9	0.38 (NS)
	End to side (N = 12)	9	3		
	Side to side (N = 9)	8	1		
NS = Not Significant;
*=Significant
